# Autonomic Involvement in Subacute and Chronic Immune-Mediated Neuropathies

**DOI:** 10.1155/2013/549465

**Published:** 2013-06-18

**Authors:** Anna Mazzeo, Claudia Stancanelli, Rita Di Leo, Giuseppe Vita

**Affiliations:** Department of Neurosciences, Azienda Ospedaliera Universitaria “G. Martino,” 98125 Messina, Italy

## Abstract

Autonomic function can be impaired in many disorders in which sympathetic, parasympathetic, and enteric arms of the autonomic nervous system are affected. Signs and symptoms of autonomic involvement are related to impairment of cardiovascular, gastrointestinal, urogenital, thermoregulatory, sudomotor, and pupillomotor autonomic functions. Availability of noninvasive, sensitive, and reproducible tests can help to recognize these disorders and to better understand specific mechanisms of some, potentially treatable, immune-mediated autonomic neuropathies. This paper describes autonomic involvement in immune-mediated neuropathies with a subacute or chronic course.

## 1. Introduction

Among the peripheral neuropathies, there is a group in which autonomic fibers are prominently or selectively targeted. Disorders of autonomic function are present when small, lightly myelinated and unmyelinated nerve fibers are affected. Signs and symptoms of autonomic involvement are related to impairment of cardiovascular, gastrointestinal, urogenital, thermoregulatory, sudomotor, and pupillomotor autonomic functions. 

Autonomic dysfunction may occur in association with various diseases such as diabetes mellitus, chronic alcoholism, amyloidosis, infections, paraneoplastic syndrome, and Sjogren syndrome (secondary autonomic neuropathies) or without any underlying disease (idiopathic or primary autonomic neuropathies) [1]. 

Sensitive and reproducible noninvasive measures of autonomic function together with laboratory and electrophysiological testing can help to establish diagnosis, allowing to recognize potentially treatable immune-mediated disorders. Tests of cardiovagal (parasympathetic), adrenergic vasoconstriction (sympathetic) are now available in most laboratories and commonly performed [2]. 

This paper will focus on autonomic involvement in immune-mediated neuropathies with a subacute or chronic course. 

## 2. Subacute and Chronic Neuropathies with Immune-Mediated Mechanisms and Autonomic Involvement 

### 2.1. Immune-Mediated Idiopathic (Primary) Autonomic Neuropathies

#### 2.1.1. Autoimmune Autonomic Ganglionopathy (AAG)

AAG includes a wide spectrum of acquired disorders characterized by diffuse autonomic dysfunction with an immune-mediated pathophysiology and positivity of ganglionic nicotinic *α*3-acetylcholine receptors (*α*3-AChR) autoantibodies. Viral upper respiratory tract infections and gastrointestinal infections have been described as antecedent infections. AAG may also be associated with vaccination, surgery, or interferon therapy. 

Classical AAG is a subacute disorder with a monophasic onset, partial spontaneous improvement, and high antibody levels (>0.5 nmol/L, normal <0.05). However, some cases of slowly progressive autonomic dysfunction may actually represent limited forms of AAG. Recently the characteristics of AAG have been reviewed, and other disorders associated with AChR antibodies including chronic forms of AAG (clinically similar to Pure Autonomic Failure), postural orthostatic tachycardia syndrome, chronic idiopathic anhidrosis, isolated gastrointestinal dysmotility, and distal small fiber neuropathy syndromes have been also discussed. All these syndromes have antibody levels lower than those seen in typical AAG with subacute onset [3]. Patients with features compatible with AAG, however, frequently have an associated malignancy, most of which are considered paraneoplastic syndromes [4]. The clinical features of AAG reflect impairment of sympathetic function (orthostatic hypotension, syncope, and anhidrosis), parasympathetic function (dry mouth, dry eyes, and impaired pupillary constriction), and enteric function (gastrointestinal dysmotility, constipation, gastroparesis, and, rarely, intestinal pseudoobstruction) [5]. Approximately 25% of AAG patients describe minor sensory symptoms, such as tingling, but objective sensory loss is not present.

Laboratory features of AAG are autoantibodies binding to neuronal ganglionic *α*3-AChR. Ganglionic *α*3-AChR are a family of ligand-gated cation channels mediating fast synaptic transmission in peripheral autonomic ganglia [6]. The serum titre of the antiganglionic AChR antibody clearly correlates with the severity of autonomic dysfunctions, and the antibody level decreases as the disease improves in AAG patients. This positive correlation between high levels of ganglionic-receptor antibodies and the severity of autonomic dysfunction suggests that the antibodies have a pathogenic role in these types of neuropathy [7]. 

In a recent study, Gibbons and Freeman demonstrated a remarkable sigmoid relationship between ganglionic AChR antibody levels and severity of orthostatic hypotension (OH), with a prominent OH when antibody levels are greater than 1 nmol/L. This observation has important treatment implications; in fact immunomodulatory therapies may fail to improve symptoms unless ganglionic AChR antibody levels are reduced below a physiological threshold [8]. 

The immune pathomechanism is confirmed by improvement after immunotherapy including immunoglobulins, plasma exchange, steroids, and other immunosuppressive drugs. Spontaneous recovery has been observed in AAG cases with acute or subacute onset; conversely, in chronic progressive cases the therapy should be initiated as early as possible before irreversible neuronal cell loss occurs [1, 9]. Immunomodulatory treatment can be effective in both seropositive and seronegative putative AAG and plasma exchange or combined therapy with immunosuppressive agents should be considered in patients who do not benefit from intravenous immunoglobulin alone [9]. After immunomodulatory treatment, a variable degree of benefit was reported in several patients with AAG. In particular, a significant improvement was observed after treatment with IVIg in one patient whose serum antiganglionic AChR antibody titer was very high [10]. Pathophysiology of seronegative forms is still unknown; however, the response in some cases to immunomodulatory treatment postulates an autoimmune mechanism [8].

Nerve conduction studies are usually normal in AAG. Sural nerve biopsy specimens show preserved myelinated fibers on light microscopy and reduction of unmyelinated fibers on electron microscopic. In a patient with slowly progressive autonomic neuropathy and positive for antiganglionic AChR antibody, sural nerve biopsy showed a reduction in the density of unmyelinated fibres with an increase in empty Schwann cell subunits and scattered collagen pockets [11]. Electron microscopic examination of sural nerve biopsy from six patients with pure autonomic neuropathy revealed a variable degree of reduction in unmyelinated fibers. The density of unmyelinated fibers tended to decrease with increasing time between the onset of autonomic symptoms and biopsy [10]. A model of AAG produced by active immunisation of rabbits against the ganglionic AChR does not exhibit morphological abnormalities of neurons in the autonomic ganglia [12].

#### 2.1.2. Enteric Neuropathies (ENs)

The enteric nervous system (ENS) represents a neural network composed of as many neurones as those detectable in the spinal cord (i.e., 80–100 million neurones). Enteric nerve cells are organized in two main plexuses, the myenteric (Auerbach's) and submucosal (Meissner's) neurones that are synaptically connected in reflex circuits. The ENS has the unique ability to control most gut functions, such as regulating secretion/absorption, vascular tone, and motility. 

A major clinical diagnostic challenge is presented by the presence of clinical syndromes such as gastroparesis or pseudoobstruction in the absence of such a systemic disease. The mechanisms leading to enteric neuropathies remain poorly understood. Pathological features described in enteric neuropathies are aganglionosis, neuronal intranuclear inclusions and apoptosis, neural degeneration, intestinal neuronal dysplasia, neuronal hyperplasia and ganglioneuromas, mitochondrial dysfunction, neurotransmitter disorders, interstitial cell pathology, and inflammatory neuropathies (cellular and humoral mechanisms) [13].

The enteric ganglionitis (EG) is an inflammatory neuropathy characterized by an inflammatory or immunological insult to the intrinsic innervation supplying the gastrointestinal tract. EG is usually associated with paraneoplastic neurological syndromes, disorders of the central nervous system, and trypanosomiasis (i.e., Chagas disease) [14–17]. Morphological pathology is characterized by diffuse lymphoid infiltration of the small intestine causing intestinal pseudoobstruction, or infiltration of myenteric ganglia causing achalasia [18, 19]. Eosinophils and neutrophils may also be present [20, 21].

Humoral mechanisms are also involved and correlate with a wide array of circulating anti-neuronal antibodies associated with an underlying disease (mainly a paraneoplastic syndrome) or with idiopathic forms of myenteric ganglionitis [4, 22, 23].

Clinical features of EG are dysmotility and delayed transit. Depending on the affected gastrointestinal tract, clinical findings include oesophageal and lower oesophageal sphincter dysmotility, gastroparesis, intestinal pseudoobstruction and colonic inertia or megacolon. In relation to the immune pathophysiology there are different reports of response to immunosuppressive treatment [24].

### 2.2. Immune-Mediated Secondary Autonomic Neuropathies

#### 2.2.1. Paraneoplastic Syndrome

Paraneoplastic neurological syndromes are rare disorders in which symptoms or signs result from damage to organs or tissues that are remote from the site of the malignancy or its metastases. The pathogenesis is thought to be immune-mediated as a result of a cross-reaction against antigens shared by the tumour and nervous system cells. Recommended diagnostic criteria for paraneoplastic neurological syndromes have been recently described by Graus and colleagues [25, 26].

Autonomic dysfunction is frequently associated with paraneoplastic disorders. Subacute sensory neuronopathy (SSN) is the most typical form of paraneoplastic neuropathy, and autonomic dysfunction has been found in some patients. In these cases, the autonomic involvement seems to predict a worse prognosis. SSN is usually associated with small-cell lung cancer and anti-Hu antibody [1]. 

In addition to the classical feature of paraneoplastic neuropathy SSN, sensorimotor neuropathies, such as Guillain-Barre' syndrome, chronic inflammatory demyelinating polyneuropathy, brachial plexopathy, and vasculitic neuropathy, are sometimes observed [27]. Paraneoplastic neuropathy can show a wide variety of symptoms ranging from sensory ataxia to painful sensory impairments. A characteristic feature of painful form patients is predominant pain symptoms, particularly mechanical hyperalgesia associated with no or only a mild degree of sensory ataxic symptoms. In these patients, the nerve fiber loss is predominant in small myelinated and unmyelinated fibers. In contrast, the ataxic form patients show a loss of predominantly large myelinated fibers. A sensory ganglionopathy that affects mainly small ganglion neurons is most likely responsible for the painful version of paraneoplastic neuropathy [28, 29]. 

Dysesthesias, lancinating pains, numbness, ataxia, areflexia, and proprioceptive loss are characteristic features of SSN. Autonomic neuropathy can be also quite disabling; symptoms include postural hypotension, gastrointestinal dysmotility, urinary retention, sicca syndrome, impotence, and pupil involvement [30].

Some paraneoplastic antibodies, such as anti-Hu and anti CV2/CRMP-5, are frequently associated with autonomic neuropathy. Antiganglionic AChR antibodies are also associated with autonomic paraneoplastic neuropathies in approximately 21% of patients [31–34]. The presence of paraneoplastic neuropathy should be considered in all patients with malignancies. Whole-body positron emission tomography/CT may be helpful to detect malignancies that cannot be detected by conventional tests. Treatment of the underlying tumor is the main therapeutic approach. Immunomodulatory therapy can be beneficial in some cases [35].

Paraneoplastic chronic gastrointestinal pseudoobstruction (CGPO) is a disorder that can be initially seen by gastroenterologists. Although it is a rare condition, clinicians should consider this differential diagnosis in otherwise unexplained gastrointestinal motor dysfunction. Associated malignancies may be small cell lung carcinoma, thymoma, gynaecological, and breast tumours. It has been hypothesized that these autoantibodies are directed against antigens shared by tumour cells and by enteric neurones (onconeural antigens, like anti-Hu, anti-VGCC, and anti-ganglionic acetylcholine receptors). Gastrointestinal symptoms usually precede tumor discovery, but not all cases have an underlying tumor [22, 23].

#### 2.2.2. Connective Tissue Diseases

Subacute and chronic autonomic dysfunctions, usually subclinical, have been described in association with connective tissue diseases including rheumatoid arthritis and mixed connective-tissue disease, systemic lupus erythematosus, scleroderma, and Sjogren syndrome. Autonomic neuropathy with symptoms of autonomic dysfunction and sympathetic and parasympathetic nervous system impairment has been well documented as an important feature of Sjogren syndrome. Severe autonomic neuropathy can be a predominant manifestation in some cases of primary Sjogren syndrome [36, 37]. The progression of autonomic disturbances is often chronic, involving both adrenergic and cholinergic systems. A wide spectrum of clinical manifestation has been reported in a large series of patients with primary Sjogren and associated neuropathy [38]. Seven forms of neuropathy including sensory ataxic neuropathy, painful sensory neuropathy, multiple mononeuropathy, multiple cranial neuropathy, trigeminal neuropathy, radiculoneuropathy, and autonomic neuropathy have been observed in 92 patients described in this study. The three patients with the autonomic form had severe autonomic symptoms with Adie's pupils, severe orthostatic hypotension with syncope in all cases, and a variable degree of hypo/an-hidrosis, abdominal pain, constipation, and diarrhea. Autonomic symptoms were often seen also in all forms of neuropathy. Autonomic symptoms are most likely related to different pathologic causes such as autonomic ganglioneuronitis and peripheral autonomic nerve involvement due to T-cell attack or ischaemia due to vasculitis as supported also by autopsy studies. Typical serological abnormalities associated with Sjogren syndrome, SS-A and SS-B antibodies, cannot be detected in many cases, so tissue biopsies should be examined in suspicious cases to make the diagnosis. 

Anecdotal reports of improved neuropathic symptoms and autonomic function following corticosteroids, immunosuppressants agents, plasmapheresis, and immunoglobulin therapy have been reported [39, 40].

Autonomic neuropathy can occur, also without dysautonomic symptoms, in mixed cryoglobulinemia, a common consequence of hepatitis C virus infection in which peripheral neuropathy has been reported in 50%–86% of patients [41]. Recently a systemic CD8+ T cell-mediated process has been demonstrated in both painful and ataxic forms of primary Sjogren's syndrome-associated neuropathy, suggesting a similar causal mechanism of cytotoxic autoimmunity to ganglion neurons [42].

#### 2.2.3. Amyloid Neuropathy

The systemic amyloidoses are a diverse group of disorders that can lead to multiorgan dysfunction through the deposition of abnormal amyloid fibrils. Various proteins have been associated with amyloidosis. Peripheral neuropathy is a common complication of many of the systemic amyloidoses and it is likely related to amyloid deposition within the nerve. Focal, multifocal, or diffuse neuropathies involving sensory, motor, and/or autonomic fibers can be observed [43]. Autonomic dysfunction is commonly associated with the polyneuropathy of both primary (AL; associated with immunoglobulin light chain) [44] and hereditary amyloidosis (Familial Amyloidotic Polyneuropathy, FAP) and less frequently with secondary amyloidosis (AA; associated with amyloid A protein). Autonomic neuropathy may affect a variety of organ systems, and different symptoms such as orthostatic hypotension, anhidrosis, erectile dysfunction, and gastrointestinal dysmotility have been reported. Symptoms may be nonspecific and this can make the diagnosis more difficult. Recognition of these conditions is important and an earlier diagnosis allows appropriate treatment when available. 

Autonomic neuropathy of unknown etiology especially if tissue amyloid deposition has been detected should be also investigated for FAP. FAP is an autosomal-dominant inherited form of amyloidosis, mostly associated with mutations of the transthyretin gene, with a prevalence higher than previously estimated. Type I (transthyretin Met30) FAP is the most common type in Japan and in Western countries. A nationwide survey of FAP TTR Met30 performed in Japan documented differences in clinical and geographic features between early- and late-onset disease. With respect to autonomic dysfunction, patients in the early-onset group showed sever symptoms, beginning in the early phase of the disease [45]. 

The ages of onset of each clinical landmark were extremely variable between patients in late-onset transthyretin Val30Met cases from nonendemic areas. Some of the patients do not manifest clinically obvious autonomic symptoms, especially in the early phase of neuropathy [46]. More than 100 mutations in TTR associated with amyloidosis have been described [47]. Phenotypic variation has been reported within the same genotype in terms of age of onset, rate of progression, and primary site of involvement. TTR-FAP usually presents itself as a progressive sensorimotor polyneuropathy, often with carpal tunnel syndrome, severe autonomic dysfunction, and cardiomyopathy. 

Decreased nerve conduction velocity, increased cerebrospinal fluid protein, negative biopsy findings, and false immunolabeling of amyloid deposits are the main causes of diagnostic errors, leading sometimes to the confusion with a CIDP. DNA testing, which is the most reliable test for TTR-FAP, should be performed in patients with a progressive length-dependent small fiber polyneuropathy of undetermined etiology, especially when associated with autonomic dysfunction [48, 49]. Therefore, detailed history taking and autonomic testing are needed to distinguish these diseases throughout the course of disease. Primary amyloid neuropathy as well as FAP may be mistaken for CIDP and may be considered in the differential diagnosis, especially in the case of poor response to immunomodulatory treatment [50]. 

Autonomic disturbances, including bowel dysfunction, postural hypotension, impotence, urinary disorders, dry skin, or truncal hyperhidrosis, may be the first signs of the disease. Recurrent syncope can be the sole manifestation of TTR-FAP for a long period of time and it must be considered in the differential diagnosis. Only by early identification of TTR-FAP can a patient benefit from an appropriate treatment [51].

Recently, Russo et al. described a cohort of patients with Leu64 mutation and late onset. At the onset of the disease, autonomic dysfunction was present in a small number of patients, but, within a few years, this had manifested in all members of the sample group. An underestimation of the disease prevalence could be caused by a late onset of FAP, which can manifest in patients up to their late 70s. The authors suggest to follow up all asymptomatic individuals to permit the early detection of symptoms and signs, allowing a detailed record of the natural history of the disease from the beginning and facilitating prompt treatment [52].

### 2.3. Chronic Inflammatory Demyelinating Polyradiculoneuropathy (CIDP)

CIDP is an immune-mediated disorder of peripheral nerves and nerve roots with a variable clinical presentation that has been considered a chronic form of Guillain-Barre' syndrome (GBS). Although it is mainly characterized by motor and sensory deficits, involvement of the autonomic nervous system (ANS) has been also reported. In contrast to GBS in which autonomic dysfunction is a common and a well-recognized complication, infrequent and mild autonomic complaints have been described in patients with chronic inflammatory demyelinating polyneuropathy (CIDP). Serious clinical dysautonomia has not been found in many large series of patients with CIDP [53–57]. Studies on dysautonomia in CIDP report variable prevalence of autonomic deficit (21% to 76%), probably due to different methods and sample size. 

Incontinence in 2% and impotence in 4% of CIDP patients have been described by Dyck et al. [58]. Prineas and McLeod reported postural hypotension in one patient and urgency of micturition in another [59]. Both invasive and noninvasive tests of autonomic function were used to investigate ANS in fourteen patients with CIDP; none of these patients complained of any autonomic symptoms. Abnormalities of the 30 : 15 ratio but normal results of other tests of vagal nerve function in 3 patients and altered thermoregulatory sweat tests in 5 patients were found suggesting mild parasympathetic and sympathetic sudomotor dysfunction. Morphometric examination of sural nerve in 10 patients showed only minor pathological changes in the unmyelinated fibers [60]. 

Subclinical autonomic dysfunction has been reported by Lyu et al. in 25% of their CIDP patients, involving both parasympathetic and sympathetic components. In the sympathetic nervous system vasomotor and sudomotor fibers may be involved. In particular, abnormal sympathetic skin response (SSR) was found in 6 of 12 patients examined [57]. 

Yamamoto and colleagues reported a case of chronic acquired neuropathy predominantly affecting sensory and autonomic nerves suggesting that autonomic nerve involvement does not exclude a diagnosis of CIDP [61]. Severe autonomic dysfunction with abnormal autonomic tests function has been reported in a chronic axonal subtype of CIDP [62].

A more extensive analysis of ANS has been performed in 17 CIDP patients showing evidence of a higher frequency of clinical and subclinical involvement than previously estimated. Six quantitative autonomic function tests were used showing abnormalities in 13 of 17 patients, mainly consistent with sympathetic and parasympathetic cardiovascular fibers impairment. Interestingly, a relatively frequent presence of clinical symptoms such as postural dizziness, erectile dysfunction, reduced foot sweating, xerostomia, and disturbances of micturition was also reported, approximating the frequency that has been reported in GBS. Dysautonomic symptoms are frequent but mild even if, upon prolonged passive standing, autonomic failure can lead to loss of consciousness [63]. 

Recently, autonomic function was retrospectively analyzed in 47 patients with classic CIDP using the Composite Autonomic Severity score that is a validated instrument for laboratory quantitation of autonomic impairment derived from standard autonomic reflexes tests. Autonomic symptoms were uncommon and when present manifested as bowel and bladder complaints. Autonomic deficits were frequent (47%) but very mild and limited to sympathetic sudomotor and cardiovagal function with relative sparing of sympathetic adrenergic function. They did not relate to duration or severity of somatic deficits and to small fiber deficits [64].

In conclusion, studies on dysautonomia in CIDP report variable prevalence of autonomic involvement. Large series of CIDP patients report mild and uncommon complaints and severe dysautonomia has been described only in anecdotal reports. 

More recent data confirm a frequent but usually very mild autonomic involvement in CIDP suggesting to consider an alternative diagnosis when an extensive or severe autonomic involvement is present. 

No autonomic complications have been reported in the subacute form of inflammatory demyelinating polyneuropathy that is a monophasic entity whose progression to nadir is between 4 and 8 weeks [65, 66].

## 3. Conclusions

Autonomic function can be impaired in subacute and chronic immune-mediated neuropathies in which sympathetic, parasympathetic, and enteric arms of the ANS are affected. Physicians should be aware of symptoms and signs suggestive of autonomic involvement, such as orthostatic hypotension, gastrointestinal dysmotility and pseudoobstruction, impotence, and urinary retention. Availability of noninvasive, sensitive, and reproducible tests can help diagnosis and management of these disorders, since some of them may cause severe complications or be life threatening. Moreover, recognition of autonomic paraneoplastic disorders can allow early diagnosis of underlying cancer. 
